# Spot Assay and Colony Forming Unit (CFU) Analyses–based sensitivity test for *Candida albicans* and *Saccharomyces cerevisiae*

**DOI:** 10.21769/BioProtoc.4872

**Published:** 2023-11-05

**Authors:** Satya Ranjan Sahu, Bhabasha Gyanadeep Utkalaja, Shraddheya Kumar Patel, Narottam Acharya

**Affiliations:** Laboratory of Genomic Instability and Diseases, Department of Infectious Disease Biology, Institute of Life Sciences, Bhubaneswar, India

**Keywords:** Spot assays, Colony forming units, Fungi, *Saccharomyces*, *Candida*, Genotoxic stress, Temperature sensitivity, DNA damaging agents, DNA replication inhibitors, Hydroxyurea, Methyl methanesulfonate, Antifungal drugs, Cell survival, DNA polymerase knockouts

## Abstract

Cellular sensitivity is an approach to inhibit the growth of certain cells in response to any non-permissible conditions, as the presence of a cytotoxic agent or due to changes in growth parameters such as temperature, salt, or media components. Sensitivity tests are easy and informative assays to get insight into essential gene functions in various cellular processes. For example, cells having any functionally defective genes involved in DNA replication exhibit sensitivity to non-permissive temperatures and to chemical agents that block DNA replication fork movement. Here, we describe a sensitivity test for multiple strains of *Saccharomyces cerevisiae* and *Candida albicans* of diverged genetic backgrounds subjected to several genotoxic chemicals simultaneously. We demonstrate it by testing the sensitivity of DNA polymerase defective yeast mutants by using spot analysis combined with colony forming unit (CFU) efficiency estimation. The method is very simple and inexpensive, does not require any sophisticated equipment, can be completed in 2–3 days, and provides both qualitative and quantitative data. We also recommend the use of this reliable methodology for assaying the sensitivity of these and other fungal species to antifungal drugs and xenobiotic factors.

## Background

Sensitivity assays are crucial in determining the susceptibility of a cell to a particular stress and are used to gain insights into diverse cellular processes in both prokaryotic and eukaryotic cells, including yeasts like *Saccharomyces cerevisiae, Candida albicans*, or *Schizosaccharomyces pombe* (Kwolek-Mirek and Zadrag-Tecza, 2014). *S. cerevisiae* and *C. albicans* are genetically related fungal species and are considered the best model organisms for sensitivity studies in yeast research due to their ease of manipulation, rapid growth, and well-established genetic modification strategies (Botstein et al., 1997; Berman, 2012). The growth curve analysis based on the optical density (OD) measurement in the presence and absence of a cytotoxic chemical is routinely used to monitor the sensitivity of a strain. Here, we describe an alternative protocol that uses a combination of spot and CFU analyses to obtain both qualitative and quantitative sensitivity results of several strains in a single experiment in a short time. We have been using this methodology repeatedly and successfully in our studies for many years now (Manohar et al., 2018; Kumari et al., 2021 and 2023; Manohar et al., 2022; Patel et al., 2023).

**Figure 1.**
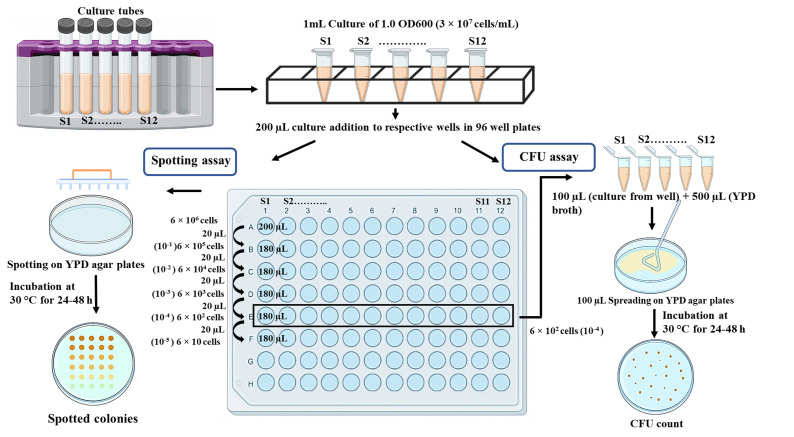
Schematic representation of the methodology to determine sensitivity of isogenic strains to a specific drug or reagent

In the spot assay, yeast cultures are spotted on agar plates with or without different concentrations of any cytotoxic agents in a dilution series; sensitivity is analyzed based on the density of the cells present in a given spot. Comparison of growth inhibition in a specific spot with and without drugs allows to assess the sensitivity of a strain to that particular drug. Similarly, relative sensitivity to a particular drug can be assessed by comparing various isogenic wildtype and mutant strains. Since spotting will only determine the cumulative qualitative behavior of a group of colonies present in a particular spot, we support this assay by enumerating colony forming unit (CFU) efficiency of a yeast strain by spreading different dilutions of the culture on solid media plates with or without different concentrations of drugs. Estimating the number of colonies and their comparison across drug concentrations or strains makes it a quantifiable sensitivity test. The combination of both assays provides us with a more comprehensive sensitivity profile of a strain. The complete assay involves spotting and spreading serially diluted yeast cells of culture on agar plates; the resulting growth patterns in both types of plates are then used to assess the sensitivity of each strain (Figure 1).

**Figure 2.**
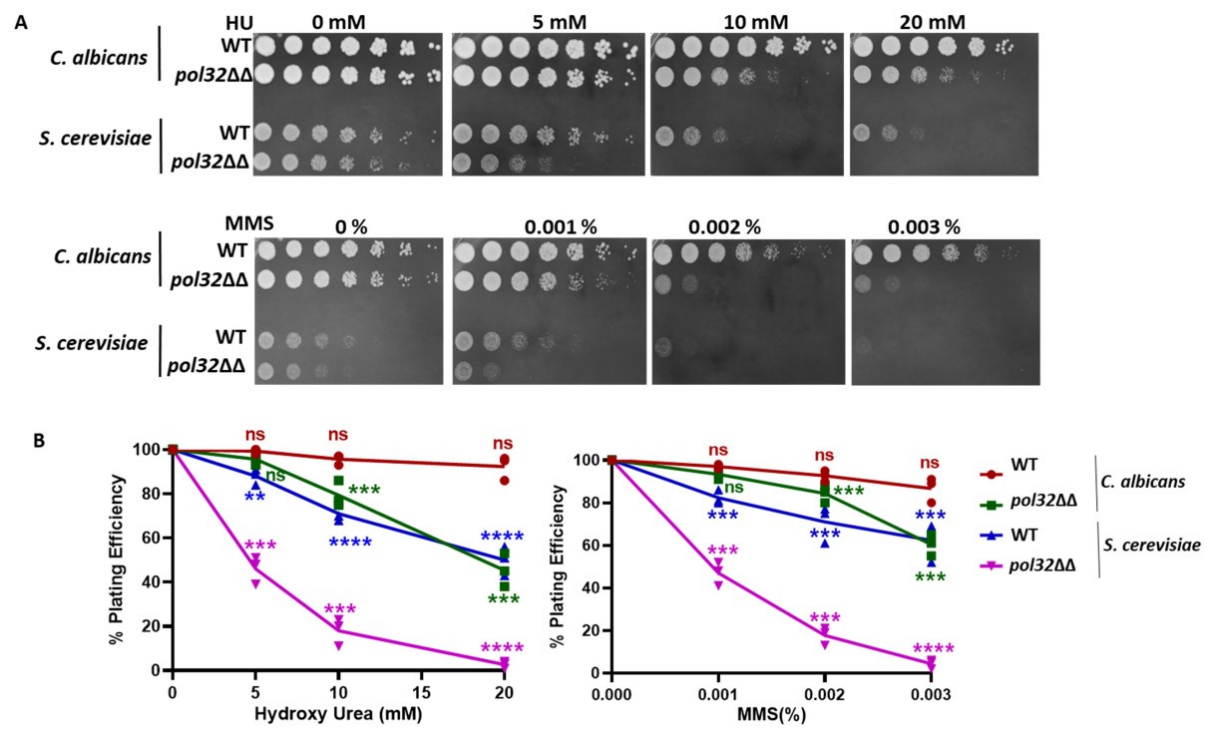
Sensitivity tests for *pol32* deficient strains of *C. albicans* and *S. cerevisiae*. (A) Cells of wildtype (WT) and *pol32* knockout strains of *C. albicans* (SC5314, diploid) and *S. cerevisiae* (EMY74.7, haploid) were spotted on YPD-agar media containing different concentrations of hydroxyurea (HU) and methyl methanesulfonate (MMS). (B) Dilutions of these cells were spread to get isolated colonies; colonies were counted and plotted in a line graph as obtained from Table 1 and Table S1. ns = nonsignificant and *** p value < 0.001.

This protocol shows the steps involved in testing four strains in their sensitivity to the presence of hydroxyurea (HU), a DNA replication inhibitor, and methyl methanesulfonate (MMS), a DNA methylating agent. However, the sensitivity of 12 different strains or drugs can be verified using a 96-well plate. We used wildtype strains of *S. cerevisiae* and *C. albicans* and their *pol32* null strains for sensitivity tests. Pol32 is the smallest subunit of DNA polymerase delta, a DNA polymerase involved in both lagging and leading strand synthesis (Acharya et al., 2011; Khandagale et al., 2019). In its absence, cells exhibit high temperature sensitivity and growth retardation in the presence of HU, MMS, and other DNA damaging agents (Patel et al., 2023). Here, we repeated the experiment to show the effect of HU and MMS on the growth of these strains by simultaneously using spot and CFU analyses (Figure 2). The differential cell density among the strains in the spot assay and the statistical estimation of the number of colonies in the CFU assay confirmed that *pol32*-deficient strains are more sensitive to these tested drugs than their respective wildtype strains.

This protocol is simple, cost effective, and does not require any expensive equipment. This assay can be used to assess multiple parameters such as growth phenotypes, viability, stress or drug resistance, and genotoxicity of multiple strains at a time (Kumari et al., 2023). Altogether, this procedure provides a well-standardized, sensitive, and reproducible protocol to assess yeasts’ sensitivity.

## Materials and reagents

1. Yeast strain of interest
2. Round-bottom glass culture tubes (Borosil, catalog number: 9900006)
3. 250 mL conical flask (Schott Duran, catalog number: 1006940)
4. Yeast extract peptone dextrose (YPD) (Himedia, catalog number: M1363)
5. Yeast extract peptone dextrose agar (YPDA) (Himedia, catalog number: G038)
6. Ethanol (100% and 70%) (Fisher Chemical, catalog number: 2051537)
7. Hydroxyurea (MP Biomedical, catalog number: 102023)
8. MMS (SRL, catalog number: 74384)
9. Sterile 1.5 mL microcentrifuge tube (Axygen, catalog number: MCT150LC)
10. Round U-bottom 96-well plate (Thermo Fisher Scientific, catalog number: 165306)
11. Sterile Petri dish 100 mm × 15 mm (Falcon, catalog number: 351008)
12. Glass Petri dish 100 mm × 15 mm (Borosil, catalog number: 3165077)
13. Sterile spreader (Tarson, catalog number: 920081)
14. Cuvettes 200–1,600 nm (Eppendorf, catalog number: 0030106300)
15. Aluminum foil (Rolias)
16. Gloves (Blue Shield)

## Equipment

1. Laminar airflow (Thermo Scientific Biological Safety Cabinets, catalog number: 41346502)
2. Sterile pipette sets (Rainin, catalog number: E1338890T-SL1000, B637036181-SL200, B641141423-SL20)
3. Laboratory spirit lamp or Bunsen burner (VWR, catalog number: 17822-605)
4. Spectrophotometer (Eppendorf, Bio Photometer Plus, catalog number: 6132)
5. 48-pin spotter (Sigma-Aldrich, catalog number: R2383)
6. Refrigerated shaker incubator (Scigenic Biotech, catalog number: LE-4676-AH)
7. Refrigerated incubator (Scigenic Biotech, catalog number: C-1NC-100)
8. Chemidoc XRS gel imager (Bio-Rad, catalog number: 1708370)
9. White Light box (Cole-Parmer: NC1851470)

## Software

1. Bio-Rad Image lab (2017,version number: 6.0.1)
2. GraphPad prism v8.0

## Procedure

These steps must be carried out in a sterile environment, preferably using a biosafety cabinet. Before starting the experiment, wipe the laminar airflow hood with 70% ethanol, place all necessary equipment (Petri dish, 96-well plates, spirit lamp, spotter, pipette set, sterile tip box, 1.5 mL MCT tubes) inside, and switch on the UV for 30 min. Use proper personal protective accessories such as laboratory jackets and gloves to prevent contamination and maintain your own safety.

- **Strain inoculation**
  1. Freshly streak all the yeast strains of interest from glycerol stock on a sterile YPD agar plate and incubate for 48 h at 30 °C.
  2. Inoculate a single colony of each strain in 5 mL of autoclaved YPD broth medium and allow it to grow overnight (14–16 h) at 30 °C in a shaker incubator at 200 rpm.
- **YPD agar plates pouring**
  1. Take freshly autoclaved YPD agar media and swirl gently until it cools enough to handle. Once you are able to hold the flask comfortably for a few seconds, pour approximately 25 mL of media per plate (see Note 1).
  2. To prepare YPD plates with HU or MMS (or any other reagents), take freshly autoclaved YPD agar media and swirl gently until it cools down to a temperature comfortable to handle. At this point, add the required volume of the chemicals’ stock solution (e.g., 100 mM HU or 99% MMS) to individual flasks, to obtain the final concentrations. Mix well by swirling and then pour. For example: for our strains, the final concentration of HU and MMS will be 5, 10, and 20 mM, and 0.001%, 0.002%, and 0.003%, respectively. A total of four plates (one for spotting and three for spreading) for each reagent concentration is required (see Note 2).
  3. Allow the plates to cool down completely for 45–60 min; this is necessary for efficient spotting and spreading. Afterward, cover the plates and keep them in an inverted position. **Critical:** Before pouring the plates with any reagents, strictly follow each toxic reagent’s user manual regarding safety, light sensitivity, thermal stability, and solubility. While pouring the plates with light-sensitive reagents, switch off the laminar hood light and cover the plates with aluminum foil. MMS and other mutagenic reagents have to be handled carefully; detoxify the flasks, plates, etc. with MMS contamination as per the supplier’s recommendation as soon as the experiment is completed. Label the plates according to the concentration before pouring the plates. Put different arrow marks to show the direction of strains and dilutions. **Pause point:** You can store plates at 4 °C by wrapping them with parafilm and aluminum foil for next-day spotting/spreading. Long-term storage is not encouraged, as the plates will dry up and the local concentration of the drugs will increase (see Additional note 1).
- **Serial dilution**
  1. Dilute the overnight grown culture to 1:10 ratio with YPD broth in a 1.5 mL microcentrifuge tube and measure the optical density (OD) in a spectrophotometer to determine culture density. **Critical:** The 600 nm OD of the undiluted culture is erroneous if it goes beyond 3; therefore, it is recommended to dilute the culture with YPD to keep OD around 1.
  2. The concentrations of different cytotoxic chemicals need to be standardized based on the strains, which may vary from experiment to experiment when more strains are included in sensitivity tests. Prior information based on published literature will be helpful.
  3. Make an equal number of cells of all strains by maintaining the OD 1.0 at 600 nm in the spectrophotometer. Double-check the OD to avoid variability in cell number.
  4. Add 200 μL of 1.0 OD culture of each strain to the first row of a 96-well plate (S1–S12); one set of samples will be for spotting and the other for the spreading CFU assay.
  5. Fill the five successive downstream wells in the columns (B1, C1, D1, E1, and F1, and similar) with 180 μL of YPD broth for serial dilution.
  6. Perform serial dilution by pipetting out 20 μL of culture from the main culture (A1) to the downstream well and continue up to the sixth well (F1) (10^-5^ dilution) for the first set of samples. By doing so, 6 × 10^6^ cells in A1 get diluted to 60 cells in F1. The same dilution will be performed for the rest of the strains (see Note 3). **Critical:** Before pipetting out 20 μL, mix it evenly for uniform cell density and efficient serial dilution.
- **Spotting of dilutions**
  1. Sterilize the spotter by dipping the pins in 100% ethanol kept in a glass Petri dish, followed by brief flaming three times. Allow the spotter to cool down (5–10 min) (see Note 4). **Caution:** Keep the spirit lamp or Bunsen burner away from the vertical airflow well inside the laminar hood. Placing the lamp too close to the airflow may cause over-flaming due to the introduction of air, which could potentially lead to an explosion of the laminar hood. Place the spirit lamp and the Petri dish with 100% ethanol in two separate corners of the laminar hood. Immediately close the lid of the spirit lamp after flaming.
  2. Once the spotter cools down completely, dip the spotter into the 96-well plate with the serial dilutions of cultures and spot gently on the agar plates with or without cytotoxic agents. Take out the spotter vertically without disturbing the plate (see Note 5). Spotting has to be done separately for each plate.
  3. After spotting, leave the plates undisturbed for 10–15 min to ensure complete absorption of spotted culture; keep the plates at 30 °C for 24–48 h depending upon the growth on the plates. **Critical:** If the additive reagent is light-sensitive, perform spotting by switching off the laminar hood light and cover with aluminum foil during incubation. After 24 h, monitor plates at regular intervals to avoid overgrowing of spots.
- **Spreading of dilutions for estimating CFU**
  1. Pipette out 100 μL of 10^-4^ diluted samples from the fifth-row wells (E1–E12), transfer into a 1.5 mL microcentrifuge tube, and make up the volume to 600 μL with YPD broth. **Critical:** Depending upon the number of plates required to spread, select the dilution accordingly from the 96-well plates (10^-3^ or 10^-4^) for further dilution in 1 mL microcentrifuge tubes. If the number of plates is > 6, one can select a lower dilution and continue.
  2. Thoroughly mix the culture in a 1.5 mL microcentrifuge tube by pipetting with a 1 mL pipette or by vortexing for a few seconds. Spread 100 μL of the resultant samples on the plates with or without reagents. Three plates containing the same concentration of HU and one YPD agar control plate will be used for spreading to get statistical significance.
  3. Incubate all plates at 30 °C for 24–48 h depending upon the growth of isolated colonies on the plates (see Additional note 2). **Critical:** Too few or too many colonies on the plates may give a false estimation; therefore, the dilution has to be selected in such a way that approximately 50–80 isolated colonies will appear on a plate. **Pause point:** Prior to imaging/counting of colonies, one can keep the plates at 4 °C by wrapping them in parafilm and aluminum foil (Figure S1).
  4. Place the plate on a light box and count the colonies manually. Note down the information with respect to each plate immediately (Additional note 3).
- **Plates imaging**
  1. Place the YPD plate from both spot and CFU analyses in an upright position on a white plate of Bio-Rad gel doc and remove the plate cover to prevent reflection. Open the Image lab software, select the *stain-free blot* in the *blot* section, and acquire the image. For best imaging, place two plates at a time (see Note 6).
  2. Export the 600 dpi image in .tif format and the raw image file in .scn format. Using any generic software (e.g., PowerPoint), arrange the spotted plates in increasing concentration order with the control plate and compare the sensitivity.

## Data analysis

1. 1 mL of yeast culture of 1 OD_600nm_ contains roughly 3 × 10^7^ cells; by taking 200 μL from that, you will obtain 6 × 10^6^ cells. Performing serial dilution up to 10^-4^ dilutions will result in 300 cells in 100 μL.
2. Diluting 100 μL further to 600 μL with YPD broth and spreading 100 μL will result in 50 cells per plate.
3. Count the colonies that appear in all the spread plates and note them down (Table 1 and Table S1). Take the number of colonies that appear on the YPD control plates (without any toxic chemical) as 100% survival and, based on that, determine the percentage of survival in the treated group as:
$$Survival\;Percentage=\frac{Total\;number\;of\;colonies\;in\;treated\;plate}{Total\;number\;of\;colonies\;in\;the\;control\;plate}\times 100$$
4. Prepare a table with the strains’ name, concentration of reagents, colony numbers, and percentages. Plot an *individual replicate with mean connected* graph from *XY family graphs* in GraphPad prism v8.0 by combining all data of biological triplicates and technical duplicates. Statistical significance will be determined by using two-way ANOVA and Tukey’s multiple-comparison test (within column, compare rows).
  **Table 1.** Representation of hydroxyurea (HU) concentrations used, the number of colonies that appeared on plates, and the respective percentage of a strain obtained during colony forming unit (CFU) analysis

*C. albicans* WT							*C. albicans pol32*ΔΔ					
**Conc. (HU)**	**Cell count**				**Average percentage (%)** ± **SD**	**P-value**	**Cell count**				**Average percentage (%) ± SD**	**P-value**
	**Rep 1**	**Rep 2**	**Rep 3**	**Avg**			**Rep 1**	**Rep 2**	**Rep 3**	**Avg**		
0 mM	75	75	75	75	100	NA	75	75	75	75	100	NA
5 mM	75	75	74	75	99 ± 0.94	0.99	70	73	72	72	95 ± 2.05	0.59
10 mM	70	73	73	72	96 ± 1.88	0.59	65	58	56	60	79 ± 4.78	< 0.001
20 mM	65	72	71	69	92 ± 4.4	0.13	29	40	33	34	45 ± 6.12	< 0.0001
***S. cerevisiae* WT**							***S. cerevisiae pol32*ΔΔ**					
**Conc. (HU)**	**Cell count**				**Average percentage (%)** ± **SD**	**P-value**	**Cell count**				**Average percentage (%) ± SD**	**P-value**
	**Rep 1**	**Rep 2**	**Rep 3**	**Avg**			**Rep 1**	**Rep 2**	**Rep 3**	**Avg**		
0 mM	75	75	75		100		74	75	75	75	100	
5 mM	63	68	66		88 ± 2.94	0.0074	31	38	36	14	46 ± 5.09	< 0.001
10 mM	53	51	56		71 ± 2.94	< 0.0001	9	17	15		96 ± 5.09	< 0.001
20 mM	20	32	38		50 ± 5.35	< 0.001	1	2	3	2	92 ± 1.24	< 0.0001

## Notes

1. Pour freshly autoclaved YPD agar media as soon as possible to avoid agar aggregation and clumping.
2. While pouring plates with chemical reagents, mix thoroughly to ensure uniform mixing of the reagent in the media and, at the same time, to avoid frothing. One has to add an appropriate concentration of the main stock of the reagent to 100 mL of YPD-agar to get the desired concentration on the plate. For example, 20 μL of 100 mM HU was added to 100 mL of media to obtain 20 mM. The stock solution of HU (100 mM) can be prepared by adding 7.6 mg to 1 mL of double-distilled water and filter sterilizing. Approximately 99% MMS is readily available with the vendor in liquid form.
3. Carefully transfer the culture from one well to the next during serial dilution to prevent spillage that might cause cross-contamination with adjacent strains.
4. Use a glass Petri plate (100 mm × 15 mm) with 100% ethanol instead of a plastic one for dipping the spotter pin before flaming.
5. During spotting, your hand should be still, avoid shivering, and gently place the spotter on the surface of the plate to avoid any damage to the solidified media.
6. While acquiring the image in a Chemidoc XRS gel imager, carefully open the plate cover and avoid touching the spots.

**Additional notes**
1. Instead of genotoxic agents, antifungal drugs and any xenobiotic compounds can be used to carry out sensitivity tests.
2. For a temperature sensitivity assay, spotted plates without any toxic reagents will be incubated at different temperatures, such as 16, 30, 37, and 42 °C.
3. Cells’ morphology from the spotting or the spread colony can be determined under a microscope to evaluate the effect of a particular stress on morphology and also to rule out any contamination during plating.

## Supplementary information

The following supporting information can be downloaded here:

1. Figure S1. YPD plates with colonies were imaged after 24 h of incubation at 30 °C before counting for CFU analysis.
2. Table S1. Representation of MMS concentrations used, the number of colonies that appeared on plates, and the respective percentage of a strain obtained during CFU analysis.
